# Spatio–temporal variation on syphilis from 2005 to 2018 in Zhejiang Province, China

**DOI:** 10.3389/fpubh.2022.873754

**Published:** 2022-08-25

**Authors:** Xiaoxia Zhu, Zhixin Zhu, Lanfang Gu, Yancen Zhan, Hua Gu, Qiang Yao, Xiuyang Li

**Affiliations:** ^1^Department of Epidemiology & Biostatistics, and Center for Clinical Big Data and Statistics, Second Affiliated Hospital, College of Medicine, Zhejiang University, Hangzhou, China; ^2^Center for Medical Science and Technology Education Development, Hangzhou, China; ^3^Department of Disease Prevention Control and Occupational Health, Zhejiang Provincial Health Commission, Hangzhou, China

**Keywords:** syphilis, spatio–temporal analysis, Bayesian spatial CAR model, epidemiological trend, Zhejiang Province, China

## Abstract

**Background:**

Syphilis has spread throughout China, especially in Zhejiang Province which endangers the health and lives of people. However, the spatial and temporal epidemiological studies of syphilis in Zhejiang are not thorough enough. The temporal and spatial variation and the relevant factors of syphilis incidence should be analyzed for more effective prevention and control in Zhejiang, China.

**Methods:**

Data on confirmed cases of syphilis in Zhejiang Province from 2005 to 2018 was used and the spatio–temporal distributions were described. The spatial autocorrelation analysis and SaTScan analysis were performed to identify spatio–temporal clusters. A Bayesian spatial Conditional Autoregression (CAR) model was constructed to explore the relationships between syphilis incidence and common social and natural indicators.

**Results:**

474,980 confirmed cases of syphilis were reported between 2005 and 2018 with a large peak in 2010. Farmers and unemployed people accounted for the largest proportion of confirmed cases. And the significant spatial clusters of syphilis were concentrated in the north of Zhejiang Province, especially in more economically developed regions. Seven spatio–temporal clusters were identified and the main three high–risk areas were located in Hangzhou (RR = 1.62, *P* < 0.05), Zhoushan and Ningbo (RR = 1.99, *P* < 0.05), and Lishui (RR = 1.68, *P* < 0.05). The findings showed that the morbidity of syphilis was positively correlated with the Gross Domestic Product (GDP) per capita, the number of health technicians per 10,000 people, the proportion of the elderly and air temperature were negatively correlated with the proportion of the urban population, the proportion of men and precipitation.

**Conclusions:**

The spatio–temporal analysis revealed that the prevalence of syphilis was still serious in Zhejiang Province. Syphilis high–risk areas were mainly located in the more developed coastal regions where more targeted intervention measures were required to be implemented. The study highlighted the need to strengthen Sexually Transmitted Diseases (STD) screening and health education for high–risk groups and improve the coverage of syphilis testing to reduce hidden syphilis cases.

## Introduction

Syphilis is a chronic and systemic disease caused by infection with Treponema pallidum through sexual and mother–to–child transmission (1). The main symptoms in the early stage of infection are hard chancre and sclerosing lymphadenitis. Without prompt treatment, syphilis can cause rash, gingivitis, cardiovascular and nervous system diseases, and even endanger life (2). More critically, syphilis is a major cause of infant mortality (3) and increases the risk of Human Immunodeficiency Virus (HIV) infection and transmission (4).

The current prevalence and incidence of syphilis among adults worldwide remain high. The number of syphilis cases was estimated to be 6.3 million (95% Confidence Interval: 5.5–7.1 million) worldwide in 2016 (5). Since the first syphilis case was reported in 1979, the number of confirmed syphilis cases has increased over time in China (6, 7). Syphilis was the fastest–growing infectious disease in China from 2004 to 2013 (8). The incidence of syphilis reported increased from 30.93/10^6^ to 38.37/10^6^, with an average annual growth of 4.41% from 2014 to 2019 (7). Economic growth, increased incidence of unsafe sex (9, 10) and improvement in syphilis screening (11, 12) were considered as potential influencing factors for this change.

Spatio–temporal analysis has been widely used to describe the geographical distribution of infectious diseases, identify high–risk spatio–temporal clusters and explore relevant variables (13), including spatial autocorrelation analysis (14) and spatio–temporal scanning analysis (15, 16). Exploring the temporal and spatial variation of syphilis can provide scientific support for public health professionals and policymakers to formulate targeted preventive measures and interventions.

Zhejiang Province is one of the provinces with a high incidence of syphilis in China (17, 18), which has ranked first among Class A and B infectious diseases in recent years (19). However, the spatio–temporal cluster distribution of syphilis cases and the correlation between the morbidity of syphilis and social and natural indicators in Zhejiang Province remain unknown. To identify epidemiological trends, priority areas and related factors of syphilis, the spatio–temporal analysis of the reported incidence of syphilis was conducted in Zhejiang Province from 2005 to 2018.

## Materials and methods

### Data collection and management

There are 90 county–level administrative regions in Zhejiang Province located in the coastal area of southeast China. Data on confirmed cases in Zhejiang Province were collected from the China Information System of Disease Prevention and Control. All cases were diagnosed and confirmed according to the Syphilis Diagnostic Standard issued by the Ministry of Health, the People's Republic of China, meeting the diagnostic criteria of clinical symptoms and laboratory evidence. Data on social and demographic indicators were collected from the China Information System of the National Bureau of Statistics. And the data on annual average temperature and precipitation were found at the European Center For Medium–range Weather Forecasts. Electronic maps of Zhejiang Province were obtained from National Fundamental Geographic Information System.

To protect patient privacy, these records were anonymized containing only gender, age, residence location, occupation, and onset date.

### Spatial autocorrelation analysis

Spatial autocorrelation was applied to analyze the spatial correlation of the incidence of syphilis in Zhejiang Province from 2005 to 2018 with the county as the spatial scale. Moran's I statistic was used at the global level and Local Indicators of Spatial Association (LISA) was for local autocorrelation (16). Positive spatial correlation meant that adjacent values in this space had similar trends while negative spatial correlation referred to the opposite (20). The diagram of LISA was drawn in local autocorrelation analysis, which identified the distribution of “high–high,” “low–low,” “low–high” and “high–low” clusters (21) of infectious disease. “Low–high” clusters meant that low values were surrounded by high values. “High–low” ones indicated that high values were surrounded by low values. Similarly, high–high clusters were significant hot spots, as well as high–risk areas of syphilis, and low–low ones meant significant cold spots (22).

### Space–time scanning analysis

Space–time scan statistic was proposed by Kulldorff in 1997 to identify the location of the space–time cluster (23). In creating a cylinder scanning window, the bottom center of the cylinder corresponded to the center of the cluster and the height of the cylinder represented the length of time for scanning. The constant change of the radius and height represented the change of the space area and period covered by the scanning window until all the space units were scanned (24). This method has been widely used in spatio–temporal cluster analysis of HIV (13), drug–resistant *Escherichia coli* (25), malaria (15) and other infectious diseases.

The spatio–temporal regions of syphilis reported cases in Zhejiang Province from 2005 to 2018 were dynamically scanned through circular scanning windows, and the Log–Likelihood Ratios (LLR) of scanning windows were calculated according to the actual values and expected values to determine high–risk clusters (26). The maximum radius threshold of the spatial window was 50% of the population at risk. The temporal cluster size of the temporal window was one day to 50% of the study period. Relative Risk (RR) in the SaTScan output file referred to the ratio of the estimated risks within and outside the cluster. A high RR indicated that people living within a cluster were at a higher risk of infection than those living outside the cluster (27).

### Bayesian spatial conditional autoregressive model

A Bayesian spatial CAR model based on Poisson distribution was modeled in this study. And indicators such as per capita GDP, the proportion of the urban population, the number of health technicians per 10,000 people (13), the proportion of people over 60 years old, the proportion of male, annual average temperature and precipitation were introduced as variables to explore related social and natural factors of the morbidity of syphilis in Zhejiang Province.

The Bayesian spatial conditional autoregressive model was a powerful estimation method of spatial effects, which aimed to update prior knowledge with new data and reduced the influence of contingent noise on new estimates under different sampling or study conditions. The prior knowledge usually referred to the intrinsic structural information and the inferred information of parameters (28). Markov Chain Monte Carlo (MCMC) algorithm was used to estimate the parameters of the model in this study (28, 29).

Observed cases (*O*_*i*_) of syphilis for the district *i* were assumed to have a Poisson distribution with mean *μ*_*i*_. The model was written as (20, 30):


(1) $$\begin{array}{r} O_{i}\sim Poisson\left(\mu_{i}\right) \end{array}$$

(2) $$\begin{array}{r} {\log\mu_{i}=logE}_{i}+\alpha_{0}+\overset{n}{\underset{j=1}{\sum }}\alpha_{j}x_{ij}+b_{i} \end{array}$$

where *i* = 1, 2, ..., 90, *E*_*i*_ meant the expected cases, based on the total incidence of syphilis in Zhejiang province in a certain year, combined with the local population distribution. α_0_ was an intercept term representing the baseline log of syphilis across the study region, *x*_*ij*_ was the variate in the district *i*, with associated regression coefficient α_*j*_ and *b*_*i*_ was an area–specific random effect capturing the residual or unexplained log RR of disease in the area *a*_*i*_. *b*_*i*_ represented the effect of latent or unobserved risk factors (20) which had spatial correlation. Winbugs14 described this model as follows:


(3) $$NormalCAR(adj_{i},w_{i},num_{i},\tau )$$

where *i* = 1, 2, ...., 90, *adj*_*i*_ was the set of neighboring regions, *w*_*i*_ was the spatial weight factor, *num*_*i*_ was the number of neighboring regions, *τ* was the reciprocal of normal variance, representing the precision. The model corresponded to the distribution of *μ*_*i*_ one by one to determine the prior distribution of *b*_*i*_. The prior distribution of other variables was as follows:


(4) $$\begin{array}{r} \alpha_{0}\sim flat\;distribution \end{array}$$

(5) $$\begin{array}{r} \alpha_{j}\sim N\left(0,10^{-5}\right) \end{array}$$

(6) $$\begin{array}{r} \tau \sim gamma\left(0.5,0.0005\right) \end{array}$$

After 10,000 iterations, the coefficients of the model tended to be stable.

### Statistical analysis methods

Continuous data with normal distribution were described by the mean and standard deviation. Continuous data with non–normal distribution were represented by the median and inter-quartile range (IQR). Classified data were described using relative numbers. Statistical graphs were drawn using Excel 2020 software. Statistical maps were drawn using R 4.1.0 software. The spatial autocorrelation analysis was conducted using GeoDa 1.18 software. The spatio–temporal cluster analysis was processed using SaTscan 9.7 software. Bayesian spatial CAR model was performed using WinBugs 14 software. The *P*–value equal to or less than 0.05 was considered statistically significant in this study.

## Results

### Descriptive analysis

**Figure 1:**
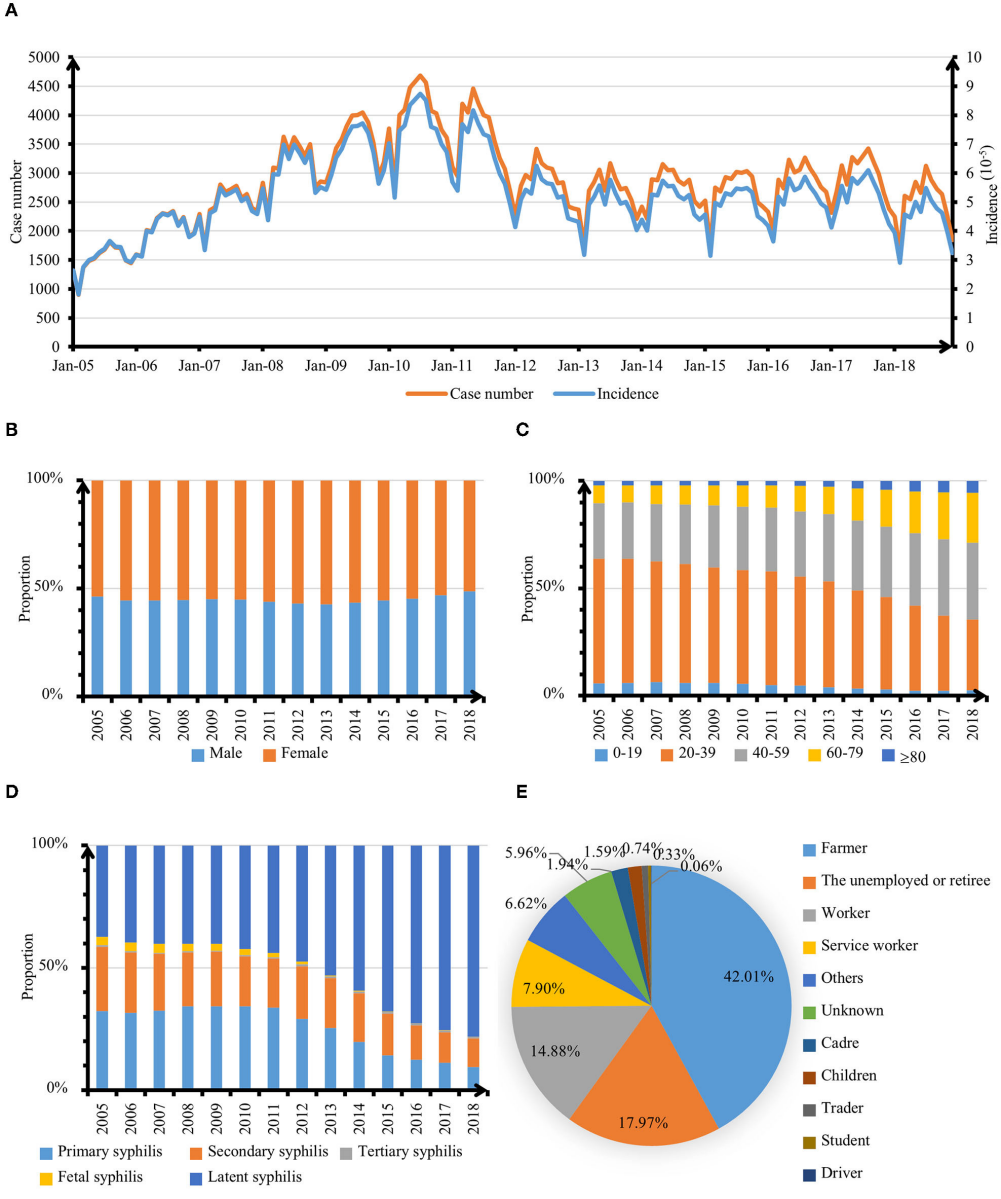
Epidemiological features of syphilis cases in Zhejiang Province, 2005–2018. **(A)** Monthly distribution of reported cases and incidence of syphilis. **(B)** Sex distribution of syphilis cases. **(C)** Age distribution of syphilis cases. **(D)** Clinical type distribution of syphilis cases. **(E)** Occupation type distribution of syphilis cases.

A total of 474,980 confirmed cases of syphilis were reported in Zhejiang Province from 2005 to 2018, covering all counties. The incidence of syphilis reported increased from 36.39/10^5^ in 2005 to 90.25/10^5^ in 2010, then dropped to 57.75/10^5^ in 2013. And it remained stable from 2013 to 2018 fluctuating between 52.72/10^5^ and 62.78/10^5^ (Figure 1A), still far higher than the average level in China (7). The number of female cases was higher than that of male cases from 2005 to 2018 and the gap between the two gradually narrowed after 2011 (Figure 1B). The average age of patients confirmed was 38 with an inter-quartile range of 25, and all the cases under 1-year-old were congenital syphilis patients (Figure 1C). For the types of syphilis, the proportion of latent syphilis was the largest from 2005 to 2018 followed by primary syphilis and secondary syphilis, and less fetal syphilis and tertiary syphilis. The number of secondary syphilis cases had gradually reached the level of primary syphilis since 2014 showing an overall downward trend while the proportion of invisible syphilis had increased rapidly (Figure 1D). Among the reported cases of syphilis, farmers accounted for the largest proportion, followed by unemployed, retired or engaged in housework, workers, and those engaged in service industries (Figure 1E).

### Spatial and temporal distribution

**Figure 2:**
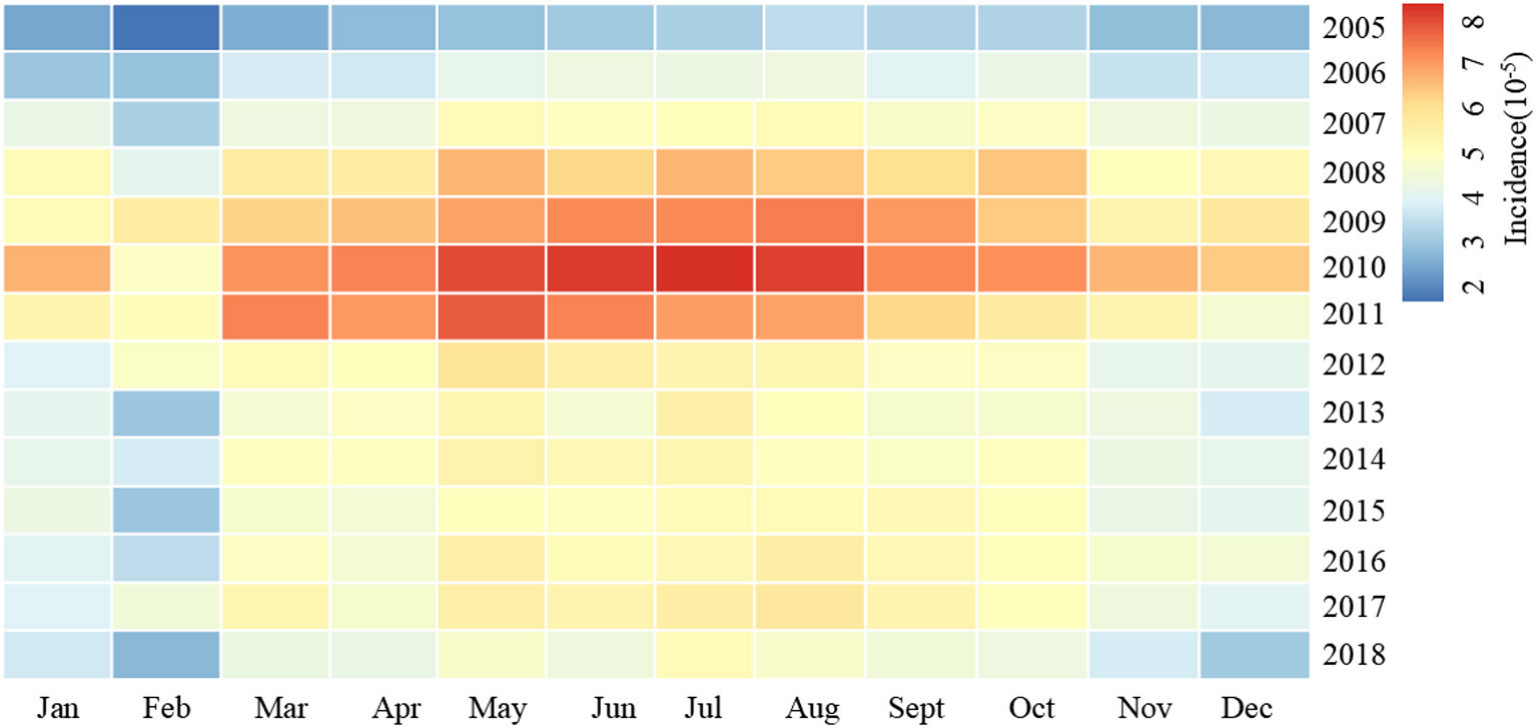
Temporal distribution of syphilis monthly incidence in Zhejiang Province, 2005–2018.

**Figure 3:**
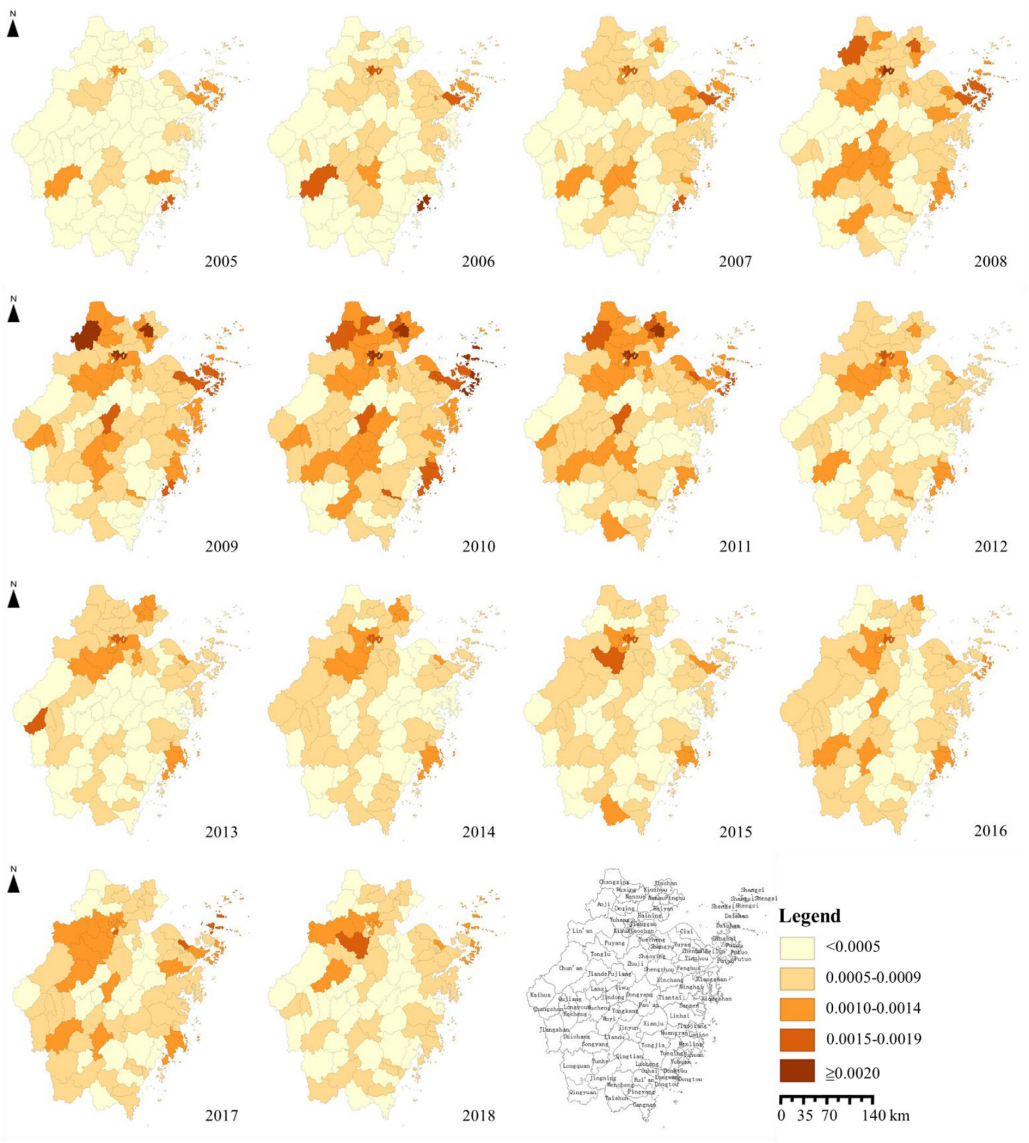
Spatial distribution of reported incidence of syphilis in Zhejiang Province, 2005–2018.

The annual incidence of syphilis increased first and then decreased in Zhejiang Province from 2005 to 2018. Seasonal patterns were identified with syphilis. The peak of monthly incidence was in May, and the trough was in February (Figure 2). The morbidity of syphilis was higher in northern Zhejiang (including Hangzhou, Huzhou and Jiaxing) and coastal areas (Ningbo and Taizhou). The incidence of syphilis was lower in the middle of Zhejiang province (Shaoxing) (Figure 3).

### Spatial autocorrelation analysis

Spatial autocorrelation analysis was conducted on the incidence of syphilis in Zhejiang province from 2005 to 2018. The global Moran's I statistic ranged from 0.18 to 0.34 (Table 1), suggesting significant positive spatial autocorrelation for syphilis incidence at the county level.

**Table 1:** Results of global spatial autocorrelation for incidence of syphilis in Zhejiang Province, 2005–2018.

**Year**	**Moran^′^s *I***	** $\bar{x}$ **	***s***	***z***	***P***
2005	0.2843	−0.0093	0.0706	4.16	0.002
2006	0.2794	−0.0099	0.0697	4.15	0.002
2007	0.3170	−0.0092	0.0710	4.59	0.001
2008	0.2339	−0.0100	0.0719	3.39	0.004
2009	0.3009	−0.0098	0.0725	4.29	0.001
2010	0.2974	−0.0096	0.0711	4.32	0.001
2011	0.3355	−0.0094	0.0709	4.68	0.001
2012	0.3129	−0.0116	0.0725	4.47	0.001
2013	0.3056	−0.0114	0.0725	4.37	0.001
2014	0.3396	−0.0109	0.0722	4.85	0.001
2015	0.2511	−0.0119	0.0729	3.61	0.001
2016	0.1799	−0.0127	0.0725	2.66	0.006
2017	0.2034	−0.0131	0.0723	3.00	0.002
2018	0.2255	−0.0111	0.0728	3.25	0.003

**Figure 4:**
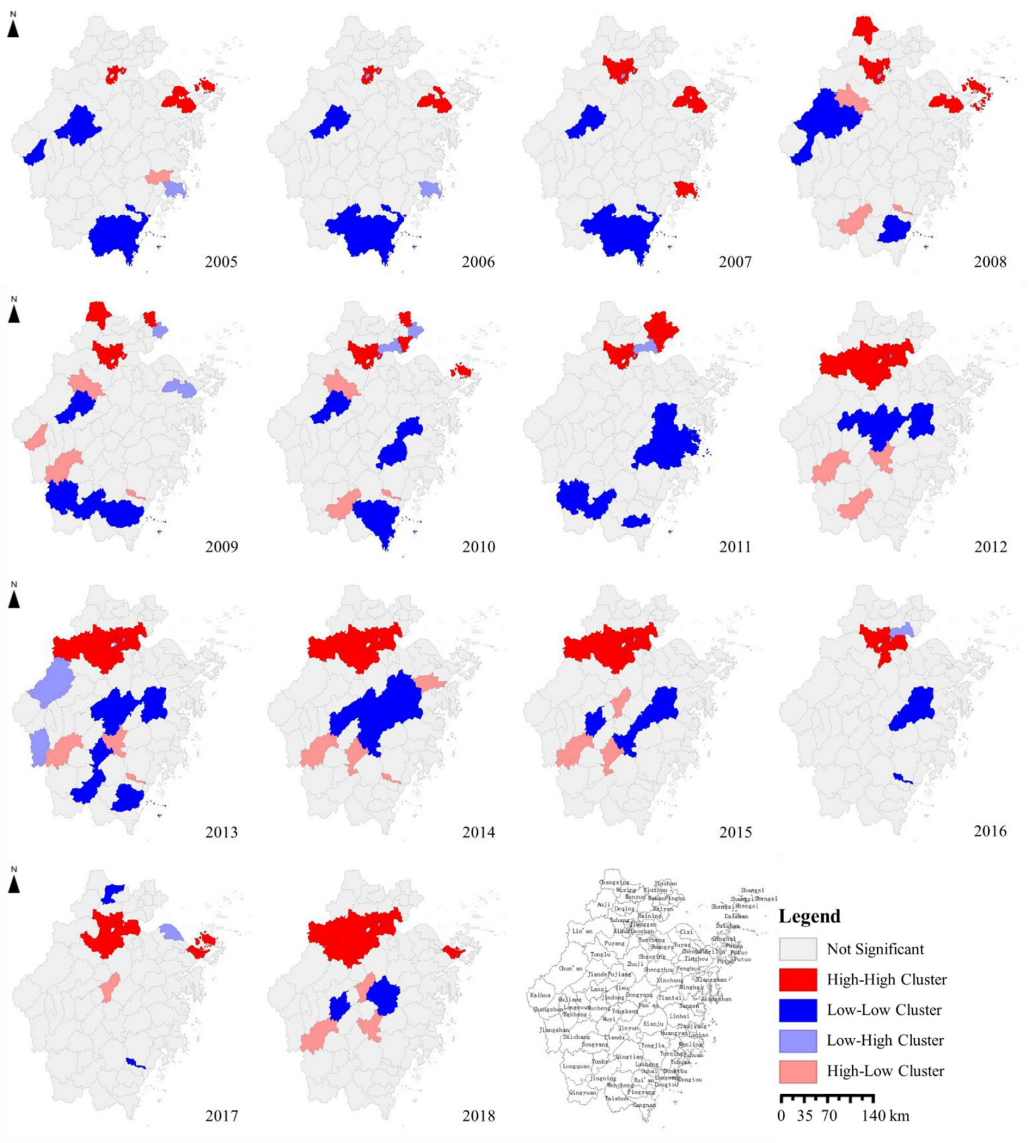
Spatial autocorrelation analysis of the reported incidence of syphilis in Zhejiang Province, 2005–2018.

The maps of LISA showed that the high–high clusters were observed in the northeast of Zhejiang Province in 2005, covering six districts in Hangzhou City (Shangcheng, Xiacheng, Xihu, Binjiang, Gongshu and Jianggan District), three districts in Ningbo City (Yinzhou, Jiangbei and Zhenhai District) and one district in Zhoushan City (Dinghai District). During the 2006–2011 period, Wenling County, Taizhou City, Yuhang District, Hangzhou City, Changxing County, Huzhou City, Putuo District, Zhoushan City, and Jiashan, Haiyan County, Xiuzhou and Nanhu District, Jiaxing City became high–high clusters successively. The northern area of Hangzhou City (Lin'an, Fuyang, Xiaoshan, Yuhang District, Haining County, and downtown, Hangzhou City) was almost covered by high–high clusters by 2015 and Beilun District, Ningbo City had also been affected since 2017 (Figure 4).

Low–low clusters, on the other hand, had shifted from the south to the middle and the coverage area reduced gradually. In 2005, low–low clusters were mainly located in Changshan County, Quzhou City, Jiande County, Hangzhou City, Lanxi County, Jinhua City and most counties of Wenzhou City (Longwan, Lucheng District, Ruian, Pingyang, Wencheng, Taishun and Cangnan County, Wenzhou City), and then moved to the south of Jinhua City and Shaoxing City from 2015 to 2018 (Figure 4).

### Space–time scanning analysis

The high–risk spatio–temporal clusters of reported syphilis cases in Zhejiang province were determined by the spatio–temporal scanning method. Seven high–risk clusters from 2005 to 2018 were identified. The most significant cluster was located in the north of Zhejiang Province, containing Hangzhou (Lin'an, Fuyang, Tonglu County, Gongshu, Shangcheng, Xiacheng, Binjiang, Jianggan and Xihu District, Hangzhou City) and Huzhou (Deqing and Anji County, Huzhou City) from September 4, 2007 to September 2, 2014. The second one covered two major coastal cities from March 30, 2005 to March 26, 2012, including Zhoushan (Dinghai, Putuo District, Daishan and Shengsi County, Zhoushan City) and Ningbo (Beilun, Zhenhai, Jiangdong, Jiangbei and Haishu District, Ningbo City). Lishui (Songyang, Suichang, Yunhe, Jinyun, Longquan, Jingning County and Liandu District, Lishui City) and and (Wuyi County, Jinhua City) were identified as the third cluster from July 20, 2005 to July 10, 2012. In addition to the three main spatio–temporal clusters, the remaining clusters were found in Taizhou (Yuhuan County and Luqiao District, Taizhou City) and most counties of Wenzhou (Table 2).

**Table 2:** Retrospective space–time scan analysis of syphilis cases in Zhejiang Province, 2005–2018.

**Dates**	**Main counties in cluster**	**(Coordinates), radius (km)**	***RR^a^***	***LLR^b^***	***P***
2007/9/4–2014/9/2	Lin'an, Fuyang, Xihu, Gongshu, Deqing, Shangcheng, Xiacheng, Anji, Binjiang, Jianggan and Tonglu	(30.23 N, 119.72 E), 48.03	1.62	4141.17	<0.001
2005/3/30–2012/3/26	Daishan, Dinghai, Putuo, Beilun, Zhenhai, Shengsi, Jiangdong, Jiangbei and Haishu	(30.25 N,122.20 E), 75.44	1.99	4121.73	<0.001
2005/7/20–2012/7/10	Songyang, Suichang, Yunhe, Liandu, Longquan, Jingning, Wuyi and Jinyun	(28.45 N,119.48 E), 61.73	1.68	1176.87	<0.001
2005/1/1–2011/12/2	Dongtou and Yuhuan	(27.83 N,121.15 E), 34.25	1.84	787.29	<0.001
2008/12/31–2015/12/21	Taishun	(27.57 N, 119.72 E), 0	1.76	340.52	<0.001
2007/5/31–2014/5/9	Luqiao	(28.58 N, 121.38 E), 0	1.43	211.08	<0.001
2008/9/29–2012/7/14	Longwan, Lucheng and Ouhai	(27.93 N, 120.82 E), 20.13	1.29	168.19	<0.001

a RR, relative risk;

b LLR, log–likelihood ratios.

### Bayesian spatial conditional autoregressive model

Based on the reported cases of syphilis in Zhejiang Province, a Bayesian spatial CAR model based on Poisson distribution was modeled, and the indicators, such as GDP per capita, the proportion of the urban population, number of health technicians per 10,000 people, the proportion of people over 60 years old, the proportion of male, annual average temperature and precipitation, were introduced as related variables to explore the social and natural factors related to syphilis epidemic.

After 10,000 iterations, the stable fitting results showed that the proportion of the urban population, the proportion of male and annual precipitation were negatively correlated with morbidity of syphilis, namely that counties with a higher proportion of the urban population, a higher proportion of male or annual precipitation had lower risk ratio. On the contrary, GDP per capita, the proportion of people over 60 years old, the number of health technicians per 10,000 people and the annual average temperature were positively associated with the morbidity of syphilis. In other words, counties with higher GDP per capita or the number of health technicians per 10,000 people had a higher risk ratio. The morbidity of syphilis incidence was relatively high in counties with a higher annual mean temperature. Areas with a higher degree of aging had a higher risk of syphilis (Table 3). It could be observed that the correlation coefficient between the two covariables of per capita GDP and health technicians and the morbidity was gradually smaller, indicating that the correlation between the two and RR decreased by degrees. This trend might be related to the regional economic development and health resources distribution in Zhejiang province gradually balanced (Table 3).

**Table 3:** Partial regression coefficient estimates and 95% confidence intervals for related social variables of syphilis in Bayesian spatial CAR model in Zhejiang Province, 2005–2018.

**Year**	**GDP per capita**	**Proportion of people over 60 years old**	**Proportion of urban population**	**Number of health technicians per 10,000 People**	**Proportion of men**	**Annual average precipitation**	**Annual average temperature**
2005	0.502 (−0.266–1.411)	0.139 (−0.138–0.471)	0.034 (−0.049–0.091)	*0.169 (0.055–0.255)*	*−0.288 (−0.417–−0.069)*	*−8.111 (−12.640–−1.691)*	*0.548 (0.113–0.917)*
2006	0.405 (−0.242–1.284)	1.164 (−0.016–1.400)	0.088 (−0.006–0.222)	0.046 (−0.051–0.130)	*−0.564 (−0.685–−0.048)*	−3.560 (−7.698– 0.101)	0.178 (−0.389–0.464)
2007	*0.799 (0.369–1.418)*	*0.665 (0.017–1.246)*	0.045 (−0.069– 0.098)	*0.099 (0.035–0.135)*	*−0.351 (−0.468–−0.053)*	*−4.934 (−9.308–−1.831)*	0.078 (−0.382–0.542)
2008	0.184 (−0.336–0.543)	*0.970 (0.156–1.496)*	−0.011 (−0.064– 0.034)	0.078 (−0.013–0.122)	*−0.485 (−0.704–−0.083)*	−0.316 (−1.864–1.914)	0.084 (−0.066–0.289)
2009	0.456 (−0.114–0.970)	0.595 (−0.060–0.913)	0.017 (−0.040–0.087)	*0.081 (0.004–0.115)*	*−0.414 (−0.509–−0.070)*	*−6.778 (−11.370–−0.625)*	*0.241 (0.044–0.519)*
2010	*0.870 (0.304–1.396)*	0.708 (−0.008–1.176)	*−0.189 (−0.247–−0.051)*	*0.070 (0.019–0.122)*	*−0.359 (−0.472– −0.047)*	*−3.916 (−6.101–−1.585)*	*0.448 (0.240–0.535)*
2011	*0.213 (0.010–0.520)*	0.058 (−0.059–0.192)	0.002 (−0.033–0.055)	*0.042 (0.003–0.079)*	*−0.140 (−0.195–−0.039)*	*−3.737 (−5.315–−1.506)*	*0.260 (0.099–0.436)*
2012	*0.183 (0.004–0.352)*	−0.135 (−0.253–0.002)	*−0.114 (−0.172–−0.031)*	*0.078 (0.017–0.101)*	−0.065 (−0.155–0.011)	1.400 (−0.980–3.739)	0.269 (−0.094–0.740)
2013	0.050 (−0.258–0.304)	0.058 (−0.189–0.307)	−0.012 (−0.091–0.036)	*0.057 (0.015–0.076)*	*−0.081 (−0.189–−0.039)*	0.440 (−1.612– 2.836)	0.177 (−0.156–0.535)
2014	−0.065 (−0.233–0.316)	−0.154 (−0.375–0.041)	0.005 (−0.070–0.051)	*0.042 (0.001–0.061)*	0.022 (−0.058–0.063)	−1.452 (−3.717–1.393)	*0.362 (0.021–0.662)*
2015	*0.284 (0.105–0.629)*	0.226 (−0.037–0.392)	−0.003 (−0.100– 0.054)	0.001 (−0.021–0.015)	−0.154 (−0.209–0.030)	*−2.737 (−5.067–−0.290)*	*0.851 (0.140–1.193)*
2016	0.180 (−0.048–0.323)	0.111 (−0.122–0.279)	0.014 (−0.042–0.050)	*0.032 (0.006–0.050)*	*−0.340 (−0.520–−0.031)*	−0.436 (−1.714–1.713)	*0.181 (0.005–0.514)*
2017	*0.165 (0.002–0.307)*	*0.653 (0.115–0.739)*	*−0.051 (−0.217–−0.004)*	0.025 (0.000–0.039)	*−0.406 (−0.461–−0.066)*	−1.700 (−3.690–1.010)	*0.187 (0.055–0.423)*
2018	0.131 (−0.054–0.312)	*0.395 (0.095–0.491)*	−0.010 (−0.099– 0.077)	*0.018 (0.002–0.035)*	*−0.333 (−0.531–−0.053)*	*−4.050 (−5.766–−0.727)*	*0.776 (0.128–1.007)*

*Italic indicates statistical significance (95% Confidence Interval does not cover zero)*.

It was noteworthy that the 95% confidence interval of the correlation coefficient between the proportion of the urban population and the morbidity of syphilis did not cover 0 only in 2010, 2012 and 2017, and even the direction of the correlation coefficient in other years often changed. This indicated that the correlation between the proportion of the urban population and the local syphilis risk ratio was not as stable as other covariables in time. The estimated values of statistically significant correlation coefficients of this covariable were all negative, showing that the proportion of the urban population was negatively correlated with risk ratio in 2010, 2012 and 2017. Likewise, the positive correlation between the proportion of the elderly and the morbidity was statistically significant only in 2007, 2008, 2017 and 2018. Therefore, the relationship between these covariables and the morbidity of syphilis needed further study (Table 3).

## Discussion

It has been found that the incidence of syphilis in Zhejiang province remains high. However, few studies used spatio–temporal analysis to analyze the prevalence of syphilis in Zhejiang Province. This study extended beyond extant literatures to reveal the high–risk clusters of syphilis in Zhejiang Province and social and natural variables that were related to syphilis morbidity of the local population. Thus, scientific support could be provided for the supervision and control of the syphilis epidemic, so as to allocate medical resources more effectively.

The number and morbidity of syphilis cases in Zhejiang province increased from 2005 to 2010 and decreased relatively during 2010–2013, which might be caused by the issuance and implementation of Syphilis Prevention and Control Plan of Zhejiang Province (2010–2020) formulated in 2012 (19). In order to reduce the incidence of syphilis, the department concerned had taken targeted measures against such problems as the occurrence of high–risk sexual behavior, low protection awareness of key groups, insufficient coverage of preventive measures and interventions, and non–standard diagnosis and treatment services of some medical institutions (31). The syphilis incidence in Zhejiang province showed seasonal variation with a low incidence in February and a peak in May, which was similar to the results of a study on syphilis in Guangdong Province, China (32). Some studies suggested that the seasonal variation may be related to environmental temperature (33). Syphilis incidence could increase with the rise of ambient temperature to some extent. The temperature might affect hormone levels and increase unsafe sex, leading to an increased burden of syphilis in the region (34). This was also consistent with the result that the local syphilis incidence risk ratio was positively correlated with the average annual temperature in this study.

Patients with latent syphilis accounted for the largest proportion from 2005 to 2018 showing an upward trend on the whole. The similar change had been observed in Guangzhou, China (35). The number of reported cases of secondary syphilis had gradually reached the level of primary syphilis with a downward trend as a whole since 2014. The increasing number of latent syphilis could be explained by extensive syphilis serum screening in medical institutions with the increase of latent syphilis detection rate. At the same time, the improvement of syphilis awareness and the initiative of counseling and testing of key groups might also be the reasons (19, 36).

The findings showed that female patients were slightly higher than male patients from 2005 to 2018. The proportion of male was also negatively correlated with the risk of developing the disease. This might be related to the fact that sex workers are generally female, and their high–risk sexual behavior leads to the high–risk group of syphilis (37). A study had shown that among drug users, women were much more likely to contract syphilis than men (38). Most patients were adults aged 20–60 especially the 20–30 age group, which was similar to the age distribution of syphilis cases in Songjiang District, Shanghai (39). And the proportion of the elderly in syphilis cases was increasing year by year. Correspondingly, the proportion of the elderly over 60 years old was positively correlated with the local syphilis incidence risk ratio. This was similar to the result of an observational study in Guangdong Province, China (from 2014 to 2015) (40). Young people, such as college students, and the elderly are exposed to a complex social environment but lack Sexually Transmitted Disease (STD) knowledge and protection awareness (41), so it is more convenient to make friends with strangers and have high–risk sexual behaviors (42). The increased risk of syphilis might be related to the increase in erectile dysfunction drugs or the increase in commercial sexual relations, suggesting that the sexual health of the elderly should be addressed by society (43). Additionally, farmers accounted for the largest proportion, followed by the unemployed and retired, workers and those engaged in service industries, which agreed with the findings of epidemiological trends and features of syphilis in China from 2014 to 2019 (7). They might be more likely to have high–risk sex for the complex social environment. Due to the fragile economic conditions and lack of knowledge about STD prevention, the limited ability of this population to obtain preventive interventions (41) has led to an increase in the incidence of syphilis. This result was confirmed by the negative correlation between the proportion of urban residents and the incidence risk ratio. Under the background of China's household registration system (44), citizens are generally divided into rural residents and urban residents. Most of the rural residents are engaged in agricultural labor, so the increase in the proportion of urban residents is similar to the decrease in the proportion of the agricultural population.

The spatial autocorrelation of syphilis incidence in Zhejiang Province revealed that the high–high clusters were concentrated in Hangzhou, Ningbo and other economically developed areas with a large population flow. The high–risk space–time regions determined by the space–time scanning were mainly concentrated in Hangzhou, Ningbo, Zhoushan and Lishui from 2005 to 2015, which was consistent with the spatial autocorrelation results.

Bayesian spatial CAR model indicated that per capita GDP and the number of health technicians showed significant positive correlations with risk ratios of syphilis, as a further explanation of high–risk clusters more concentrated in large cities than small ones. One of the reasons might be that major population movements triggered by economic growth have increased the risk of developing infectious disease (17, 45). Another contributor to the distribution of clusters could be the social networks in developed economies or coastal areas and the entertainment industry have developed rapidly. Men who have Sex with Men (MSM) (46), sex workers (47) and other high–risk groups are sexually active generally so that they are more prone to have high–risk sexual behaviors or drug injection, which cause the spread of syphilis and other infectious diseases. Furthermore, it may be related to the inequitable allocation of health resources, which could give rise to the difference in syphilis diagnosis and monitoring ability in different cities. Higher syphilis diagnosis accuracy and monitoring coverage will lead to higher syphilis detection rates in big cities (17, 40). On the other hand, the Bayesian analysis also suggested that the proportion of the urban population, the proportion of men and annual precipitation were negatively correlated with the risk ratio of syphilis, consistent with the results of the above descriptive epidemiology analysis. There were few studies on the relationship between syphilis incidence risk ratio and precipitation. It might be related to high rainfall limiting people's travel and reducing the frequency of high–risk contacts. Low level of education (48), poor health awareness of rural population (41) and weak basic public health services of rural areas (49, 50) might result in that rural population is a high–risk group for syphilis infection. Therefore, while strengthening urbanization, the improvement of rural infrastructure and the health education of farmers reduced local syphilis incidence to some extent.

A limitation of this study is that people may not be proactive in syphilis testing due to the “stigmatization” of STDs including syphilis (12, 17). And Bayesian spatial CAR model was built to analyze the correlation between various factors and the incidence of syphilis. Further research is needed to pin down causation. The Bayesian spatial model for the study of the factors related to the morbidity of syphilis only considered the spatial effect. In further research, time variables should be included to make the model more perfect.

The population characteristics and temporal and spatial changes of confirmed syphilis cases in Zhejiang Province from 2005 to 2018 were revealed in this study. The results suggested that local health departments needed to strengthen health management, screening and interventions for high–risk groups such as sex workers, farmers, unemployed and floating populations. At the same time, schools and education departments could improve the publicity and education on the knowledge of prevention and treatment of STDs among the elderly and college students. This study revealed that the risk of the syphilis epidemic was higher in areas with a developing economy and a large population flow. The locations of high–risk clusters such as Hangzhou and Ningbo needed attention and the change in syphilis incidence in real time should be tracked to implement targeted intervention strategies and provide better preventive services. In addition, the incidence of syphilis reported is also related to local health resources. Therefore, increasing the coverage of syphilis testing is necessary to reduce the underreporting of syphilis cases. In conclusion, exploring high–risk clusters and the characteristics of key groups gain insight into the spread of infectious diseases to develop preventive measures and interventions.

## Data availability statement

The original contributions presented in the study are included in the article/supplementary material, further inquiries can be directed to the corresponding authors.

## Author contributions

XL and XZ designed the research study. XZ performed the research and wrote the manuscript. QY, HG, XZ, ZZ, LG, and YZ provided help and advice on data collection. All authors contributed to editorial changes in the manuscript, read, and approved the final manuscript.

## Funding

This research was funded by the Soft Science Key Project of the Science and Technology Department of Zhejiang Province (2022C25040), and the Soft Science Key Project of Hangzhou Municipal Science Committee (20160834M03). Thanks to all the peer reviewers and editors for their opinions and suggestions.

## Conflict of interest

The authors declare that the research was conducted in the absence of any commercial or financial relationships that could be construed as a potential conflict of interest.

## Publisher's note

All claims expressed in this article are solely those of the authors and do not necessarily represent those of their affiliated organizations, or those of the publisher, the editors and the reviewers. Any product that may be evaluated in this article, or claim that may be made by its manufacturer, is not guaranteed or endorsed by the publisher.
